# Night eating and night eating syndrome: associations with dysfunctional eating behaviors, mental health and quality-of-life measures in Australian adults

**DOI:** 10.1007/s40519-025-01732-5

**Published:** 2025-03-13

**Authors:** Haider Mannan, Stephen Touyz, Phillipa Hay

**Affiliations:** 1 THRI, Western Sydney University, Campbelltown, NSW Australia; 2 Inside Out Institute, University of Sydney, Camperdown, NSW Australia; 3 Mental Health Services, South Western Sydney Local Health District, Campbelltown, NSW Australia

**Keywords:** Night eating, Night eating syndrome, Binge-eating, Purging, Mental health, Quality of life

## Abstract

**Purpose:**

The association of night eating (NE) and NE syndrome (NES) with dysfunctional eating behaviors, mental health and quality-of-life outcomes has been little explored in the general population. The objective of this study was to explore this for dysfunctional eating behaviors: binge-eating, use of purging, dietary restriction, use of medication to control weight; mental health: anxiety/depression; and quality-of-life outcomes: mental and physical health-related quality of life (M/PHRQoL). NE captured whether in the past 3 months, the respondents had any episodes of waking from sleep and eating, or episodes of eating a very large amount of food after evening meal excluding any such events at social gatherings or travelling overseas on a night flight or because of work shifts. NES was defined by at least weekly episodes of NE with ‘a lot’ of distress.

**Methods:**

In 2017, 2977 adults from randomly selected households in South Australia were interviewed. Analyses for bivariate association were conducted using weighted tetrachoric and weighted polychoric correlations, and ordinal and binary logistic models, to determine the association between current (3 months) NE or NES as an outcome, and binge-eating, use of purging, dietary restriction, use of medication to control weight, anxiety/depression, mental and physical health-related quality of life (M/PHRQoL) as predictors after controlling for age, sex, and body weight. All analyses adjusted for design effect by stratified cluster sampling.

**Results:**

Ordinal logistic regression found significantly higher odds of episodes of NE with binge-eating (OR = 1.756, 95% CI 1.527–2.020, *p* < 0.001), and significantly lower odds with increased MHRQoL (OR = 0.948, 95% 0.921–0.975, *p* < 0.001) and increased PHRQoL (OR = 0.976, 95% CI 0.966–0.986, *p* < 0.001). Binary logistic regression found significantly higher odds of NES with binge-eating (OR = 2.62, *p* < 0.001), and restrictive dieting (OR = 2.491, 95% CI 1.647–3.769, *p* < 0.01), and significantly lower odds with MHRQoL (OR = 0.913, 95% CI 0.879–0.948, *p* < 0.001).

**Conclusions:**

Those with a history of binge-eating have higher likelihood of having both NE and NES which are also increased for the former in those with poorer MHRQoL and PHRQoL, and for the latter in only those with poorer MHRQoL. Revisions of diagnostic schemes may consider these findings in the context of delineation of boundaries between eating disorder syndromes.

**Levels of evidence:**

Multivariate binary logistic regression analyses found there were significantly higher odds of having night eating syndrome in association with binge eating and restrictive dieting and significantly lower odds of night eating syndrome in association with increases in MHRQoL. These results support the Muscatello et al. (Aust N Z J Psychiatry 56:120–1362022, 2022) review noting associations and overlap between night eating syndrome and other eating disorders characterized by recurrent binge-eating, and the reported associations with disorders of restrictive eating. As both night eating and binge-eating are symptoms of over or excessive eating this was not unexpected. However, the findings at a diagnostic level in this study did also support research that has found overlap between night eating syndrome and disorders characterized by restrictive eating and/or purging behaviors. By “at a diagnostic level” what we meant was when at a level associated with marked distress, as the DSM requires either functional impairment or psychological distress to be present as a defining feature of any mental health disorder and distress is a defining feature of NES (American Psychiatric Association 2013). Diagnostic and Statistical Manual of Mental Disorders: DSM-5. Arlington, American Psychiatric Publishing Inc.). MHRQoL rather than PHRQoL was associated with night eating syndrome. More research is required to confirm this result and it does not negate the clinical importance of consideration of physical health status of people with night eating syndrome (Muscatello et al. Aust N Z J Psychiatry 56:120–136, 2022; Sakthivel et al. Eat Weight Disorders-Stud Anorexia Bulimia Obes 28:77, 2023). This study did not find that those who have perceived subjective anxiety/depression have significantly higher odds of NE as well as NES. This may have been because the present study did not have an assessment of depression or anxiety using a validated instrument, but rather a broad self-reported experience of current perceived anxiety and/or depression. Other studies have been also more often conduced in clinical populations which may be expected to have higher rates of mental health comorbidities (Muscatello et al. Aust N Z J Psychiatry 56:120–136, 2022) than this general population sample.

**Public significance statement:**

To our knowledge this is the first study in a representative adult general population examining the relationships between night eating (NE), NE syndrome (NES) and binge eating, purging, strict dieting and general anxiety or general depression. Those with a history of binge eating and having poorer MHRQoL and PHRQoL have higher likelihoods of experiencing NE. The same associations of these factors except for that of PHRQoL were found with NES. Revisions of diagnostic schemes may consider these findings in the context of delineation of boundaries between eating disorder syndromes. As the nature of overeating is defined more broadly in NES than in other eating disorders it is important to explore all forms of overeating when undertaking estimates of the population prevalence and burden of eating disorder.

**Supplementary Information:**

The online version contains supplementary material available at 10.1007/s40519-025-01732-5.

## Introduction

Night Eating (NE) is defined in the Diagnostic and Statistical Manual of Mental Disorders-5 (DSM-5) as excessive food consumption after the evening meal (evening hyperphagia) or eating after awakening from sleep (nocturnal ingestions) and it is the key clinical feature of NE Syndrome (NES). NES is a type of Other Specified Feeding or Eating Disorder (OSFED) in the DSM-5 [1].

The majority of research on NES has been conducted within clinical and high weight settings, where frequent comorbidity with other eating disorders and physical health problems have been reported [2]. Mitchison et al. [3] has reported NES to be particularly common (estimated prevalence 2.7%) in a representative sample of adolescents. They also found it was the most common OSFED in adolescent boys, more so than for girls. A representative study of adults, however, found NES to be more prevalent in women than in men [4]. This study also reported high levels of socioeconomic disadvantage, sleep problems, body weight, and poorer physical health-related quality of life (PHRQoL) relative to others in the general population including people with other eating disorders.

However, there has been a paucity of research in representative adult general population samples examining the relationships between NE or NES and dysfunctional eating disorders, mental health and quality-of-life measures. In addition, there is an incomplete understanding of the relationships between NE or NES and other eating disorders, such as binge eating and use of laxatives, and avoidant or restrictive feeding intake disorders (ARFID), such as restrictive dieting to control shape/weight and use of drugs to reduce appetite/overeating, as well as food avoidance or restriction for other reasons as is found in Avoidant/Restrictive Food Intake Disorder (ARFID). Furthermore, the majority of literature is from clinical samples, and that the relationship between NES and other key eating disorders and eating disorder features remains to be elucidated for a representative sample of adult population. These behaviours particularly binge eating and restrictive dieting are known to be increasing in a representative adult population sample, when co-morbid with high weight, and are a public health concern [5].Studies using clinical samples have found subjects diagnosed with NES more likely to have a comorbid eating disorder, whose prevalence ranges from 5% to 44% [6–8]. In another clinical study, NES was detected in 10.3% of patients with AN, in 34.9% of patients with BN and in 51.7% of patients with BED [9]. Regarding the association between NES and health-related quality of life (HRQoL) using a representative general adult population sample, one study [10] has found a negative association between the two. The present study thus examined the associations of these clinical features (perceived subjective anxiety or depression, mental health-related quality of life (MHRQoL), PHRQoL, binge eating, restrictive dieting to control shape/weight, use of laxatives, use of drugs to reduce appetite/overeating and restrictive dieting to control shape/weight), with NE or NES, as an outcome variable in a representative adult population. For the purpose of the study, the DSM-5 descriptor of NES was used as it was impracticable to include all the proposed criteria by Allison et al. [11] in a large community epidemiological sample. These included questions on whether in the past 3 months, the respondents had any episodes of waking from sleep and eating, or episodes of eating a very large amount of food after evening meal excluding any such events at social gatherings or travelling overseas on a night flight or because of work shifts. NES was defined by at least weekly episodes of NE with ‘a lot’ of distress.

## Materials and methods

### Study design, sampling, and weighting

We used data as in the previously reported study [4] from the 2017 Health Omnibus Survey (HOS) of people aged 18 years and over, representing the South Australian population. It used multistage sampling for which cities and towns were initially stratified into Metropolitan and Country statistical areas (SA) based on the 2016 census, and 398 metropolitan and 132 regionals were selected with a probability of selection proportional to their size. Furthermore, a skip pattern of every fourth household, was utilized to select ten households within each level 1 SA and only one structured interview was conducted per household between September and December 2017. In instances where there was more than one eligible resident, the respondent was the participant with the most recent birthday. From the 5,300 households selected, 2977 interviews were conducted, with a response rate of 57.0% and a participation rate (after 6 attempts at contact) of 65.3%.

The interview included questions pertaining to demographics and health, e.g., medical conditions, weight control etc. Verbal consent was obtained for adult participants and written consent was obtained from parents or guardians of participants under the age of 18 years. In addition, missing data were obtained by telephone interview and weighted by Australian 2016 Census data on age, sex, marital status, educational attainment, country of birth and household income. This study reports data from the 2905 participants aged ≥ 18 years.

### Measures

*Demographics* Sociodemographic data were collected via interview and weighted by Census data on age, sex, marital status, educational attainment, country of birth and household income. Participants self-reported their height and weight, and BMI (kg/m^2^) was calculated.

*Current eating disorder and mood symptoms* The “eating disorders” being assessed were not diagnosed with a standardized measure but with only symptoms which were measured using a standardized interview questions derived from the Eating Disorder Examination [12]. Participants were asked regarding DSM-5 episodes of NE, episodes of binge-eating in the last 3 months, the presence of distress and other emotions associated with these episodes, purging (vomiting and laxative use) in the last 3 months and strict dieting/fasting for weight/shape control in the last 3 months. In this study, NES was defined by at least weekly episodes of NE with ‘a lot’ of distress. The question to determine NE asked whether in the past 3 months, the respondents had any episodes of NE, where by NE, it meant waking from sleep and eating, or episodes of eating a very large amount of food after evening meal excluding any such events at social gatherings or travelling overseas on a night flight or because of work shifts. The frequency of night and binge eating were characterised by, “not at all”, “less than weekly”, “once a week”, “two or more times a week”, “don’t know” or “refused”. Participants responded to questions regarding distress associated with binge eating and night eating with the responses: “not at all”, “yes—a little”, or “yes—a lot” or with “refused”. The presence of at least weekly purging, strict dieting/fasting and a lifetime history of appetite/overeating reducing medication use, were categorised by “yes” or “no”. Levels of perceived subjective anxiety (PSA) and perceived subjective depression (PSD) were rated on a 5-point Likert scale from none to extreme (see Additional file 1 for the interview questions).

Physical and mental health-related quality of life (P/MHRQoL): Participant health status was assessed via the validated Short Form 12-item health-related quality of life (SF12) tool [13]. From the 12-items, two subscales are derived, a Physical Component Summary Scale (PCS) and a Mental Component Summary Scale (MCS). Each scale has a mean score of 50 and standard deviation of 10, with higher scores indicating an increased HRQoL.

### Ethics

The 2017 HOS was approved by the University of Adelaide Human Research Ethics Committee (HREC) with Ethics Approval ID: H-097-2010. An exemption from the HREC review was obtained for data analyzed and published beyond 2019 on the condition that the data sets are fully de-identified prior to being shared with researchers that were not included on the original ethics approval and analysis aligns with consent provided by participants.

### Data analysis

To perform bivariate associations of the ordinal variable episodes of NE or NES with binary variables use of purging in the last 3 months, lifetime use of drugs to reduce appetite/overeating, diet/fasting to control shape in the last 3 months, we used weighted tetrachoric correlation, and with ordinal variables binge eating episodes in the last 3 months (four categories), perceived subjective anxiety/depression (five categories), and with continuous variables MHRQoL and PHRQoL, we used weighted polychoric correlation and weighted polyserial correlation, respectively, while adjusting for stratified cluster sampling in all. The significance of correlation was tested by Rao–Scott methods. We used ordinal logistic regression (proportional odds model) adjusted for stratified cluster sampling, to determine the link between the outcome NE or NES and the following covariates: binge eating episodes in the last 3 months, use of purging and lifetime use of drugs to reduce appetite/overeating, diet/fasting to control shape in the last 3 months, perceived subjective anxiety/depression, mental and physical health-related QOLs, after controlling for age, sex, and body weight. The controls were included to account for potential confounding by these variables. In the regression analyses, the ordinal covariates were treated as continuous. Since missing data were negligible (< 2%), they were not imputed while performing statistical analysis. All analyses were performed using R version 4.2.2 (for bivariate associations) and SAS 9.4 [14].

## Results

There were 1196 (41.17%) males and 1709 (58.83%) females in the sample. Mean age and body weight were 48.33 (SE = 0.345) years and 79.07 kg (SE = 0.3609), respectively. These descriptive statistics of demographic characteristics are not shown in any table. However, Table 1 shows the descriptive statistics of the covariates of interest.

**Table 1:** Descriptive statistics and ordinal logistic regression of episodes of NE as the dependent variable, on other eating disorder symptoms, perceived subjective anxiety/depression and MHRQoL and PHRQoL after adjusting for the effects of age, sex and body weight

Covariate	Descriptive statistics	Dependent variable Episodes of NE		
Categorical covariate	*N* (%)	OR (95% CI)	SE	*T* statistic
Use of purging in the last 3 months	15(0.52%)	1.893 (0.497–7.213)	0.3363	0.95
Restrictive dieting/fasting for weight/shape control in the last 3 months	129(4.44%)	1.270 (0.743–2.169)	0.1346	0.89
Lifetime use of medications to reduce eating/weight	136(4.68%)	1.884 (0.872–4.069)	0.1937	1.64
Continuous covariate	Mean (SE)			
Binge-eating in the last 3 months	1.34 (0.0144)	1.756 (1.527–2.020)	0.0703	8.02*
Perceived Subjective Anxiety/Depression	1.28(0.0121)	1.170 (0.895–1.528)	0.1345	1.17
MHRQoL	51.96(0.1717)	0.948 (0.921–0.975)	0.0143	− 3.74*
PHRQoL	48.18(0.1949)	0.976 (0.966–0.986)	0.0052	− 4.64*

*p < 0.001; SE indicates standard error. The effects of control variables age, sex and body weight are not shown

One hundred and sixty-seven (5.75%, 95% CI 4.9–6.6%) adults (aged 18–99 years) had NES. As DSM-5 does not specify exclusion criteria for OSFED, the estimated prevalence of OSFED—NES in this sample was 49(1.69%, 95% CI 1.7–41.7%).

Correlations between the episodes of NE and other symptoms and HRQoL are found in Additional file 2 (Table S1). Table1, also presents using ordinal logistic regression, the associations of MHRQoL, PHRQoL, binge-eating in the last 3 months, purging in the last 3 months, lifetime use of drugs to reduce appetite/overeating, diet/fasting to control shape in the last 3 months and perceived subjective anxiety/depression, with the odds of episodes of NE (*n* = 167), after controlling for age, sex and body weight. Compared to no NE at all, there were 1.756 times significantly higher odds (OR = 1.756, 95% CI 1.527–2.020, *p* < 0.001) of NE, with binge-eating episodes in the last 3 months. There were 5.2% and 2.4% significantly lower odds of NE in association with MHRQoL (OR = 0.948, 95% CI 0.921–0.975, *p* < 0.001) and PHRQoL (OR = 0.976, 95% CI 0.966–0.986, *p* < 0.001) indicating that the association was stronger with MHRQoL in terms of the effect size.

Table 2 presents using binary logistic regression the associations of MHRQoL, PHRQoL, binge-eating episodes in the last 3 months, purging in the last 3 months, lifetime use of drugs to reduce appetite/overeating, diet/fasting to control shape in the last 3 months and perceived subjective anxiety/depression, with the odds of having NES (*n* = 49), after controlling for age, sex and body weight. There were significantly lower odds of NES in association with MHRQoL (OR = 0.913, 95% 0.879–0.948, *p* < 0.001), indicating that those who had better MHRQoL were 8.7% less likely to have NES. Compared to no NES at all, there were significantly higher odds of NES, with binge-eating episodes in the last 3 months (OR = 2.458, 95% CI 1.982–3.047, *p* < 0.001), indicating that those with binge-eating were 2.458 times more likely to have NES. The odds of NES were 2.491 times higher (OR = 2.491, 95% CI 1.647–3.769), for those dieting to control shape in the last 3 months compared to those who did not, the effect reached the significance level of 1% (*p* < 0.001). Other symptoms did not have significant association with NES.

**Table 2:** Binary logistic regression of NE syndrome (yes vs no) as the dependent variable, on other eating disorder symptoms, perceived subjective anxiety/depression and MHRQoL and PHRQoL after adjusting for the effects of age, sex and body weight

Covariate	Dependent variable NES (yes vs no)		
Categorical covariate	OR (95% CI)	SE	*T* statistic
Use of purging in the last 3 months	1.213 (0.380–3.871)	0.2918	0.33
Fasting/dieting to control shape in the last 3 months	2.491 (1.647–3.769)	0.1041	4.38*
Lifetime use of medications to reduce eating/weight	1.826 (0.547–6.098)	0.3032	0.99
Continuous covariate			
Binge-eating episodes in the last 3 months	2.458 (1.982–3.047)	0.1081	8.32*
Perceived subjective anxiety/depression	1.389 (0.882–2.187)	0.2283	1.44
MHRQoL	0.913 (0.879–0.948)	0.0189	− 4.84*
PHRQoL	0.998 (0.973–1.024)	0.0129	− 0.15

*p < 0.001; SE indicates standard error. The effects of control variables age, sex and body weight are not shown

## Discussion

The present study investigated associations of NE and NES with binge-eating, weight control behavior, including use of purging, dietary restriction and medication, mood symptoms and HRQoL. Binge eating, use of purging, dietary restriction, medication, mood symptoms and HRQoL were captured by the following variables: episodes of binge-eating in the last 3 months, use of purging in the last 3 months, strict dieting/fasting for weight/shape control in the last 3 months, lifetime use of medications to reduce eating/weight, perceived subjective anxiety/depression, MHRQoL and PHRQoL. When adjusting for age, sex and body weight, multivariate ordinal logistic regression analyses found there were significantly higher odds of an increase in episodes of NE, in association with binge-eating episodes and significantly lower odds of increase in episodes of NE, in association with increases in MHRQoL and PHRQoL. With similar adjustments for age, sex and body weight, multivariate binary logistic regression analyses found there were significantly higher odds of having NES, in association with binge eating episodes in the last 3 months and restrictive dieting in the last 3 months and significantly lower odds of NES, in association with increases in MHRQoL. These results support the Muscatello et al. [2] review noting associations and overlap between NES and other eating disorders characterized by recurrent binge-eating, and the reported associations with disorders of restrictive eating. As both NE and binge-eating are symptoms of over or excessive eating, this was not unexpected. However, the findings at a diagnostic level in this study did also support research that has found overlap between NES and disorders characterized by restrictive eating and/or purging behaviors. By “at a diagnostic level” we meant at a level associated with marked distress, the DSM [1] requires either functional impairment or psychological distress to be present as a defining feature of any mental health disorder and distress is a defining feature of NES. Refinements of diagnostic criteria may address this by closer alignment of the descriptors of overeating behaviors and clarification of the boundaries between NES and other eating disorders.

Mental rather than physical health-related quality of life is associated with NES. More research is required to confirm this result and it does not negate the clinical importance of consideration of physical health status of people with NES [2, 4, 15]. This study did not find that those who have anxiety/depression have significantly higher odds of NE, as well as NES. This may have been because the present study did not have an assessment of depression or anxiety using a validated instrument, but rather a broad self-reported experience of current anxiety and/or depression. Other studies have been also more often conduced in clinical populations which may be expected to have higher rates of mental health comorbidities [2] than this general population sample. However, often self-reported anxiety is close to population prevalence rates and is, therefore, appropriate as a quick assessment of likely anxiety. While the use of self-reported depression (SRD) in health surveys and cohorts may be appropriate as a brief assessment of possible depression, due to its moderate–low sensitivity in reference to a validated tool like PHQ-8 for depression [16]. Importantly, SRD has high global agreement (92.7%) with PHQ-8; still the prevalence of depression was higher when assessed with self-reported measurement (7.7%) than with PHQ-8 (5.9%) [16]. The association between perceived subjective anxiety/depression, and NE/NES, may thus have been biased in this study due to the use of self-reported measurement for anxiety/depression.

Although this study included a moderate sized, community-based, adult sample, there were limited participants with diagnostic level NES and thus analyses were also done at the level of NE as a symptom. The potential bias of self-reported data could not be excluded; however, the validity of self-reported data has been supported by several studies and is commonly utilized [17, 18]. Trained lay persons rather than clinicians conducted the interview as is usual in large epidemiological surveys; however, 10% of interviews were audited to ensure fidelity to the interview questions. (This was thus a respondent-based interview not investigator based). Finally, as the study is cross-sectional, causality and bidirectionality could not be investigated.

Notwithstanding these limitations, the present findings have clinical relevance. NES and NE should be considered and asked about in people presenting with other eating disorders, particularly but not limited to disorders of recurrent binge-eating and in those with poorer mental and/or physical health-related quality of life. As the nature of overeating is defined more broadly in NES than in other eating disorders, it is important to explore all forms of overeating, including whether there is loss of control, and not assume NE is absent in people who do not report binge eating.

## Conclusion

Those with a history of BE have a higher likelihood of experiencing NE and NES. The likelihood of NE is also increased in those with poorer mental and physical health-related quality of life. The same is found for the association between NES and mental health-related quality of life. Longitudinal research is required to further investigate the associations identified in this paper, particularly whether they are bi- or uni-directional.

## Supplementary Information

Below is the link to the electronic supplementary material.

Supplementary Material 1.

Supplementary Material 2.

## Data Availability

No data sets were generated or analysed during the current study.
