# Supplementary material for: Night eating and night eating syndrome: associations with dysfunctional eating behaviors, mental health and quality-of-life measures in Australian adults
Source: Eat Weight Disord. 2025 Mar 13;30(1):24. doi: 10.1007/s40519-025-01732-5 (PMC11906568; doi:10.1007/s40519-025-01732-5)
Supplement: Supplementary file 1 — Supplementary Material 1. [file 40519_2025_1732_MOESM1_ESM.docx]

**Additional File 1**

**Interview questions used in the present study.**

**Note: prompts are in brackets and instructions in italics.**

Changing the subject. I would now like to ask you about episodes of overeating. By overeating, or binge eating, I mean eating an unusually large amount of food in one go and at the time feeling that your eating was out of control. (Prompt: Respondent could not prevent themselves from overeating,or could not stop eating once they had started).

G1 Over the past three months how often have you overeaten? Would you say…

1 Not at all *Go to G6*

2 Less than weekly

3 Once a week

4 Two or more times a week

5 Don’ t know *Go to G6*

6 *Refused* *Go to G6*

G2 Is the binge or overeating you experience usually associated with distress?

1 Not at all

2 Yes – little

3 Yes – a lot

4 *Refused*

G3 Is the binge or overeating you experience usually associated with? *Multiple response Show prompt card G1*

1 Eating much more rapidly than normal

2 Eating until feeling uncomfortably full

3 Eating large amounts of food when not feeling physically hungry

4 Eating alone because you are embarrassed about how much you are eating

5 Feeling disgusted, guilty or very depressed after eating

6 None of these

7 *Refused*

The next questions are about various weight-control methods some people use.

G8 Over the past three months have you regularly used, that is at least once a week, any of the following: laxatives, diuretics (water tablets), made yourself sick, in order to control your shape or weight?

1 Yes

2 No

3 *Refused*

G9 Over the past three months have you regularly done any of the following: gone on a very strict diet, or eaten hardly anything at all for a time, in order to control your shape or weight? (At least once weekly, or recurrently during the three months.)

1 Yes

2 No

3 *Refused*

G10 On a scale of 0-6, where 0 is Not at all important and 6 is Extremely or the most important issue. How important an issue has your weight and/or your shape been to how you think about (judge or view) yourself as a person in the past three months? (Prompt: It has been a really important issue to them, their self-esteem or their self-confidence).

*Enter number or (R) for refused*

G11 In the past 3 months have you had any episodes of night eating? By night eating I mean waking from sleep and eating (i.e. you were not sleep walking and eating, you were awake), OR episodes of eating a very large amount after your evening meal. (This does not include eating at night because of social or other circumstances e.g., you are travelling overseas on a night flight or because of work shifts) Would you say you are night eating…

1 Not at all *Go to G13*

2 Less than weekly

3 Once a week

4 Two or more times a week

5 Don’ t know *Go to G13*

6 *Refused Go to G13*

G12 Is this night eating you experience usually associated with distress?

1 Not at all

2 Yes – little

3 Yes – a lot

4 *Refused*

G13 Have you ever (in your lifetime) regularly used or been prescribed drugs to reduce your appetite and/or reduce overeating or binge eating? (e.g. Duromine, an amphetamine, topiramate, Topamax, orlistat, Xenical)

1 Yes

2 No

3 *Refused*

**Question regarding anxiety/depression.**

Which of the following statements best describes your health TODAY regarding….

… O8. Anxiety/Depression? *Show prompt card O5*

1 I am not anxious or depressed

2 I am slightly anxious or depressed

3 I am moderately anxious or depressed

4 I am severely anxious or depressed

5 I am extremely anxious or depressed
