# Supplementary material for: Night eating and night eating syndrome: associations with dysfunctional eating behaviors, mental health and quality-of-life measures in Australian adults
Source: Eat Weight Disord. 2025 Mar 13;30(1):24. doi: 10.1007/s40519-025-01732-5 (PMC11906568; doi:10.1007/s40519-025-01732-5)
Supplement: Supplementary file 2 — Supplementary Material 2. [file 40519_2025_1732_MOESM2_ESM.docx]

**Table S1. Associations between the episodes of night eating and binge eating, purging, restrictive diet to control weight/shape, use of drugs to reduce eating, mental health and quality of life variables for study participants aged 18 years and over**

| Variable | Correlation | Standard Error | Rao-Scott/Wald Statistic |
| --- | --- | --- | --- |
| Binge eating in the last 3 months | 0.42 | 0.038 | 21.81*** |
| Use of purging | -0.29 | 0.117 | 3.85 |
| Use of drugs to reduce eating | -0.20 | 0.059 | 29.52** |
| Restrictive diet to control weight/shape | -0.30 | 0.059 | 1.50 |
| Anxiety/depression | 0.41 | 0.038 | 27.83*** |
| MHRQoL | -0.30 | 0.026 | 129.00* |
| PHRQoL | -0.06 | 0.034 | 2.84 |

Note: * p<0.05, ** p<0.01, ***p<0.001; Rao-Scott test used for all variables except for MHRQoL and PHRQoL, Wald test used for MHRQoL (Mental Health Relate Quality of Life) and PHRQoL (Physical Health Relate Quality of Life.
